# Maternal Haemodynamic Changes During Spinal Anaesthesia for Caesarean Section

**DOI:** 10.7759/cureus.108159

**Published:** 2026-05-03

**Authors:** Nidhi Agrawal, Sonal Solanki, Vacha J Patel, Yogesh S Kirwale

**Affiliations:** 1 Obstetrics and Gynaecology, Government Medical College, Datia, Datia, IND; 2 Obstetrics and Gynaecology, Sumandeep Vidyapeeth (Deemed to be University), Vadodara, IND; 3 Anaesthesia, Dr. N.D. Desai Faculty of Medical Science and Research Centre, Dharmsinh Desai University, Nadiad, IND; 4 Anaesthesia, Government Medical College, Datia, Datia, IND

**Keywords:** caesarean section, hypotension, mean arterial pressure, spinal anaesthesia, vasopressor

## Abstract

Background

Spinal anaesthesia is widely preferred for elective caesarean sections because of its rapid onset and safety advantages. However, sympathetic blockade frequently results in maternal hypotension, which may compromise uteroplacental perfusion. Despite preventive measures, hypotension remains common, and reliable preoperative predictors of haemodynamic instability are limited.

Objective

This study aimed to assess maternal mean arterial pressure (MAP) changes following spinal anaesthesia, determine the incidence and temporal pattern of hypotension, and examine the association between early MAP decline (ΔMAP) and vasopressor requirement.

Methods

This prospective observational study included 100 parturients undergoing elective caesarean section under spinal anaesthesia at the Department of Obstetrics and Gynaecology and Anaesthesia at the associated hospital of Government Medical College, Datia, Madhya Pradesh, India. Demographic and obstetric characteristics were recorded. Haemodynamic parameters were measured at baseline and at serial intervals up to 120 minutes. Hypotension was defined as systolic blood pressure <90 mmHg or a ≥20% reduction from baseline. The primary outcome was the change in MAP (ΔMAP) within the first 30 minutes. Serial comparisons and multivariable logistic regression analyses were performed to evaluate temporal trends and associations.

Results

The mean age was 27.4 ± 4.6 years, and the baseline MAP was 91.9 ± 3.8 mmHg. Hypotension occurred in 78 (78%) participants, and 34 (34%) required vasopressors. MAP declined significantly at five and 10 minutes post-spinal (p ≤ 0.003), indicating early haemodynamic compromise, followed by recovery after 15 minutes. The mean reduction from baseline to minimum MAP was 20.3 mmHg (p < 0.001). Age, BMI, gravidity, and baseline MAP were not significantly associated with hypotension. Greater reductions in MAP (ΔMAP) were significantly associated with vasopressor requirement (adjusted odds ratio (OR) 3.6, 95% confidence interval (CI) 1.90-6.78; p < 0.001).

Conclusions

Spinal anaesthesia is associated with frequent early hypotension. Greater declines in MAP were associated with increased vasopressor use, reflecting the severity of haemodynamic compromise rather than an independent predictive factor. These findings provide descriptive insights into perioperative haemodynamic patterns in routine clinical practice.

## Introduction

Spinal anaesthesia is the preferred technique for elective caesarean sections because it provides a rapid onset of dense sensory blockade, reliable surgical conditions, reduced airway manipulation, and improved maternal safety compared with general anaesthesia. Despite these advantages, spinal anaesthesia provokes a predictable sympathetic blockade that leads to peripheral vasodilation, reduced systemic vascular resistance, and subsequent maternal haemodynamic instability. Consequently, maternal hypotension remains the most frequent complication associated with this technique and continues to pose significant perioperative concerns.

The occurrence of hypotension after spinal anaesthesia has been widely reported in obstetric populations, with an incidence ranging from 50% to 80% in routine practice [1]. In the absence of prophylactic measures, the incidence may increase further, reaching 70-80% [2]. Reviews of perioperative obstetric anaesthesia indicate that spinal-induced hypotension is common, often abrupt, and clinically relevant because it may cause maternal nausea, vomiting, dizziness, decreased uteroplacental perfusion, and potential fetal compromise [3]. Contemporary literature similarly emphasises that this problem persists even with modern monitoring and management strategies, necessitating prompt detection and intervention [4].

Although hypotension is universally recognised, there is considerable inconsistency in its definition and measurement. Variable thresholds based on systolic blood pressure, mean arterial pressure (MAP), or the percentage decline from baseline have led to wide heterogeneity in reported incidence. Therefore, a consensus-based approach has been proposed, recommending maintaining systolic blood pressure above 90% of baseline and MAP above 70 mmHg, with vasopressor therapy initiated at lower thresholds to prevent clinically significant compromise [5]. The lack of standardised definitions complicates comparisons across studies and highlights the need for objective haemodynamic indices, such as MAP and time-based monitoring.

Several strategies have been investigated to prevent or attenuate spinal-induced hypotension. Fluid therapy alone has shown limited efficacy, particularly crystalloid preloading, which often fails to prevent vasodilation-induced hypotension [6]. Earlier studies have demonstrated that although rapid crystalloid or colloid administration may reduce the incidence to some extent, no single fluid strategy reliably eliminates haemodynamic instability [7]. Systematic reviews and meta-analyses have shown that interventions such as crystalloid or colloid loading, ephedrine, lower-limb compression, and their combinations may reduce the frequency of hypotension but remain insufficient as standalone measures [8].

Consequently, vasopressor therapy has emerged as the cornerstone of the management of these patients. Contemporary evidence supports prophylactic and therapeutic vasopressor use as the most effective strategy for maintaining maternal blood pressure during caesarean delivery under spinal anaesthesia [9]. Phenylephrine, an α-adrenergic agonist, has increasingly become the first-line agent because of its effectiveness in restoring vascular tone and its association with improved fetal acid-base status compared with that of ephedrine [10]. Meta-analyses suggest that although both drugs are similarly effective at correcting hypotension, phenylephrine is associated with higher neonatal pH values and fewer maternal adverse effects [11]. More recently, norepinephrine infusion has been proposed as an alternative with additional β-adrenergic activity and potentially improved haemodynamic stability [12]. Continuous prophylactic infusions and automated closed-loop systems have demonstrated superior blood pressure control compared with intermittent bolus administration, further emphasising the importance of early and sustained haemodynamic management [13,14]. Despite advances in prophylaxis and treatment, maternal hypotension remains highly prevalent in diverse settings. Reports indicate that even with modern vasopressor protocols, hypotension may occur in 38-75% of cases [15,16].

Many studies have focused on prevention strategies and interventions rather than characterising haemodynamic changes after spinal anaesthesia. The literature from high-resource centres provides limited real-world data on haemodynamic trends and predictors of instability in routine practice. The evaluation of MAP trajectories and factors associated with vasopressor requirements remains to be explored. Understanding haemodynamic profiles and identifying high-risk patients are important for optimising monitoring and ensuring maternal safety. Changes in MAP provide objective perfusion measures compared with systolic blood pressure alone. Evaluating the associations between patient characteristics and intervention needs may help in risk stratification. 

The primary objective of the study is to describe temporal changes in MAP following spinal anaesthesia in elective cesarean section. The secondary objectives are to estimate the incidence and timing of hypotension and examine the association between early MAP decline (ΔMAP) and vasopressor requirement. This study focuses on real-world perioperative haemodynamic patterns rather than intervention efficacy.

## Materials and methods

Study design and setting

This prospective, hospital-based observational study was conducted at the Department of Obstetrics and Gynaecology and Anaesthesia at the associated hospital of Government Medical College, Datia, Madhya Pradesh, India. This study was designed to evaluate maternal haemodynamic changes following spinal anaesthesia in parturients undergoing elective caesarean section and to identify the incidence and factors associated with spinal anaesthesia-induced hypotension and vasopressor requirement.

Study population and sample size

A total of 100 pregnant women were consecutively recruited. A formal sample size calculation was not performed. The sample size was based on feasibility and was considered adequate for estimating the incidence of hypotension and for exploratory regression analysis of associated factors in a perioperative obstetric population. Participants were recruited consecutively to minimise selection bias.

Inclusion Criteria

Pregnant women scheduled for elective lower-segment caesarean section under spinal anaesthesia, aged 18-40 years with a singleton term pregnancy and classified as American Society of Anesthesiologists (ASA) physical status I or II, were included.

Exclusion Criteria

Women with hypertensive disorders of pregnancy, cardiovascular or endocrine comorbidities, multiple gestation, emergency caesarean section, contraindications to spinal anaesthesia, or refusal to participate were excluded to avoid confounding haemodynamic alterations.

Preoperative preparation and anaesthetic technique

All participants underwent routine pre-anaesthetic evaluation and fasting, as per the institutional protocol. Baseline haemodynamic parameters were recorded in the supine position prior to spinal block. Intravenous access was secured, and crystalloid preloading of 10-15 mL/kg was performed. Co-loading was not used, in accordance with institutional practice. No prophylactic vasopressor infusion was administered.

Spinal anaesthesia was performed under strict aseptic precautions in the sitting position at the L3-L4 or L4-L5 interspace using a 25G or 26G Quincke needle. After confirming free cerebrospinal fluid flow, 2.0-2.5 mL of 0.5% hyperbaric bupivacaine was intrathecally injected. Immediately after the procedure, patients were positioned supine with left uterine displacement to minimise aortocaval compression. Sensory block height was assessed clinically but was not included in the analysis.

Intraoperative management protocol

Hypotension was managed using intravenous vasopressors (primarily phenylephrine or ephedrine, depending on availability). Bolus doses were administered when systolic blood pressure decreased below 90 mmHg or by ≥20% from baseline. Repeat dosing was guided by clinical response. No standardised vasopressor infusion protocol was used.

Haemodynamic monitoring and data collection

Continuous intraoperative monitoring was available using a multiparameter monitor, including noninvasive blood pressure, heart rate, and peripheral oxygen saturation. However, data were recorded at predefined intervals (baseline and at two, five, 10, 15, 20, 30, 60, and 120 minutes) for analysis.

Demographic and clinical variables, including age, height, weight, body mass index, and gravida, were documented. Systolic and diastolic blood pressures were measured at each time point, and MAP was calculated using the standard formula:

$MAP= \frac{(SBP+2*DBP)}{3}$

MAP was calculated manually from recorded systolic and diastolic blood pressure values.

Outcome variables and operational definitions

The primary outcome variable was the change in MAP from the baseline to the lowest value recorded within the first 30 minutes following spinal anaesthesia. The minimum MAP was defined as the lowest MAP observed during this interval, and the difference between the baseline MAP and this value was calculated as ΔMAP. Hypotension was defined as a systolic blood pressure of less than 90 mmHg or a reduction of at least 20% from baseline. Bradycardia was defined as a heart rate of less than 60 beats per minute. The requirement for therapeutic intervention was defined as the administration of vasopressors, intravenous fluids, or atropine to treat haemodynamic instability. All episodes were managed in accordance with institutional protocols. If vasopressors were administered before reaching the lowest MAP, the minimum MAP recorded prior to intervention was used to calculate ΔMAP. Haemodynamic instability was operationally defined as hypotension (systolic blood pressure <90 mmHg or a ≥20% reduction from baseline) and/or bradycardia (heart rate <60 beats per minute) that required therapeutic intervention.

Statistical analysis

Data were entered into Microsoft Excel (Microsoft Corp., USA) and analysed using jamovi statistical software (version 2.6.44, the jamovi Project, Sydney, Australia, retrieved from https://www.jamovi.org). Continuous variables are expressed as mean ± standard deviation or median (interquartile range), and categorical variables are presented as frequency and percentage. Normality of distribution was assessed using the Shapiro-Wilk test.

For the primary objective, the baseline MAP was compared with the minimum MAP using a paired t-test or Wilcoxon signed-rank test, as appropriate. Serial haemodynamic changes over time were evaluated using repeated-measures analysis of variance with a Greenhouse-Geisser correction to account for the violation of sphericity, and effect sizes were reported using partial eta-squared. Post-hoc comparisons with Bonferroni adjustment were used to identify significant differences between time points. The incidence of hypotension and vasopressor requirement was calculated as proportions. To identify factors associated with hypotension and the need for intervention, a binary logistic regression analysis was performed, and results were expressed as adjusted odds ratios (ORs) with 95% confidence intervals (CIs) and Wald chi-square statistics. Multicollinearity was assessed using the variance inflation factor, and model fit was evaluated using the Hosmer-Lemeshow test. ROC analysis was initially planned but not included in the final analysis due to limited model discrimination. Statistical significance was set at p < 0.05. No missing data or dropouts were observed during the study period.

Ethical considerations

Institutional ethical approval (letter no. 158/ANESTH/GMC/IECBMHR/2024) was obtained from the Institutional Ethics Committee of Biomedical and Health Research in Human Participants (IECBMHR) prior to the commencement of the study. Written informed consent was obtained from all participants, and the confidentiality of patient data was maintained throughout the research process.

## Results

The study included 100 pregnant women who underwent elective caesarean sections with spinal anaesthesia. The study group was relatively young, with an average age of 27.4 ± 4.6 years, and their mean height and weight were 160.8 ± 5.9 cm and 72.3 ± 9.8 kg, respectively. The mean body mass index (BMI) was 28.0 ± 4.3 kg/m², indicating that most of the participants were overweight. The average gravida was 2.0 ± 0.8, suggesting a predominantly multigravida status. Baseline haemodynamics were stable, with a mean systolic blood pressure (SBP) of 118.3 ± 5.2 mmHg, diastolic blood pressure (DBP) of 78.4 ± 4.1 mmHg, and MAP of 91.9 ± 3.8 mmHg. Despite these normal preoperative parameters, 78 (78%) participants experienced intraoperative hypotension, and 34 (34%) required vasopressor support, indicating a high incidence of spinal anaesthesia-induced haemodynamic instability (Table 1).

**Table 1:** Baseline demographic, obstetric, and haemodynamic characteristics of study participants (N = 100) Values are expressed as mean ± standard deviation (SD) for continuous variables and as frequencies with percentages for categorical variables. Hypotension is defined as intraoperative blood pressure falling below the predefined study threshold. Vasopressor requirement indicates patients who require pharmacological support to maintain blood pressure during the procedure. BMI: body mass index; SBP: systolic blood pressure; DBP: diastolic blood pressure; MAP: mean arterial pressure

Variable	Value
Age (years)	27.4 ± 4.6
Height (cm)	160.8 ± 5.9
Weight (kg)	72.3 ± 9.8
BMI (kg/m²)	28.0 ± 4.3
Gravida	2.0 ± 0.8
Baseline SBP (mmHg)	118.3 ± 5.2
Baseline DBP (mmHg)	78.4 ± 4.1
Baseline MAP (mmHg)	91.9 ± 3.8
Hypotension, n (%)	78 (78%)
Vasopressor required, n (%)	34 (34%)

A comparison of serial MAP measurements showed similar baseline haemodynamics between groups, with no significant difference at T0 (92.2 ± 3.14 vs. 91.8 ± 4.00 mmHg; p = 0.758). Early post-spinal readings at two minutes also remained comparable. However, significant divergence emerged at five and 10 minutes, where the hypotension group exhibited a greater decline in MAP than the stable group (72.8 ± 4.67 vs. 76.5 ± 4.24 mmHg; mean difference 3.67 mmHg, p = 0.003 at five minutes, and 72.8 ± 5.11 vs. 77.4 ± 3.60 mmHg; mean difference 4.33 mmHg, p < 0.001 at 10 minutes). This period corresponded to the maximal haemodynamic compromise. No significant differences were observed from 15 minutes onward, indicating that hypotension was transient in patients who did not require vasopressors, whereas patients requiring vasopressors experienced more significant haemodynamic compromise necessitating intervention (Table 2). These differences reflect the operational definition of hypotension and should be interpreted as descriptive of group classification rather than indicative of independent physiological divergence.

**Table 2:** Comparison of mean arterial pressure at serial time points according to the hypotension status (n = 100) Values are presented as mean ± standard deviation. “No hypotension” indicates participants who maintained haemodynamic stability; “Hypotension” indicates participants who developed hypotension intraoperatively. The mean difference represents the absolute difference between group means. U denotes the test statistic. time points represent minutes after spinal anaesthesia. MAP: mean arterial pressure

Time point	No hypotension (n = 22); mean ± SD (mmHg)	Hypotension (n = 78); mean ± SD (mmHg)	Mean difference (mmHg)	U	p-value
Baseline (T0)	92.2 ± 3.14	91.8 ± 4.00	0.33	821	0.758 NS
2 min	84.2 ± 3.69	83.6 ± 4.74	0.67	812	0.705 NS
5 min	76.5 ± 4.24	72.8 ± 4.67	3.67	501	0.003**
10 min	77.4 ± 3.60	72.8 ± 5.11	4.33	396	<0.001***
15 min	83.9 ± 3.27	83.5 ± 4.47	0.33	812	0.705 NS
20 min	90.2 ± 2.79	89.8 ± 4.30	0.33	815	0.723 NS
30 min	91.6 ± 3.37	91.2 ± 4.19	0.33	819	0.749 NS
60 min	92.0 ± 3.12	91.4 ± 4.10	0.33	814	0.714 NS
120 min	91.9 ± 3.29	91.2 ± 4.02	0.67	771	0.469 NS

A significant reduction in MAP was observed following spinal anaesthesia. The mean decrease between the baseline MAP and the minimum MAP recorded within the first 30 minutes was 20.3 mmHg (p < 0.001), indicating clinically meaningful hypotension.

Regression analysis did not identify age, BMI, gravida, or baseline MAP as significant factors associated with hypotension, suggesting that baseline demographic or obstetric factors alone were insufficient to explain variability in haemodynamic response. By contrast, ΔMAP was significantly associated with vasopressor requirement (adjusted OR, 3.6 (95% CI, 1.90-6.78); p < 0.001). However, this association reflects the severity of post-spinal haemodynamic changes rather than serving as an independent predictor, since ΔMAP is derived from the same physiological event that prompts intervention. Other variables, including age, BMI, and gravida, were not significant. These findings indicate that the magnitude of blood pressure decline, rather than baseline characteristics, is associated with the need for intervention (Table 3). This association reflects the severity of haemodynamic change rather than a causal or predictive relationship.

**Table 3:** Regression analysis of factors associated with maternal hypotension and vasopressor requirement Values represent multivariable logistic regression estimates. β denotes regression coefficient (log odds), SE denotes standard error, and χ² denotes Wald chi-square statistic. Adjusted odds ratios (OR) are presented with 95% confidence intervals (CI). ΔMAP represents the reduction in mean arterial pressure from baseline to the minimum value within the first 30 minutes after spinal anaesthesia. A p-value < 0.05 was considered statistically significant. **p < 0.01, ***p < 0.001. NS: not significant, MAP: mean arterial pressure, BMI: body mass index

Variable	β	SE	χ²	Adjusted OR	95% CI	p-value
Hypotension						
Intercept	1.063	6.76	0.02	2.89	—	0.875 ^NS^
Age	−0.034	0.055	0.38	0.97	0.87–1.08	0.542 ^NS^
BMI	0.101	0.061	2.74	1.11	0.98–1.25	0.095 ^NS^
Gravida	−0.172	0.324	0.28	0.84	0.45–1.59	0.596 ^NS^
Baseline MAP	−0.014	0.069	0.04	0.99	0.86–1.13	0.838 ^NS^
Vasopressor requirement						
Intercept	−19.131	5.933	10.41	4.9×10⁻⁹	—	0.001**
ΔMAP	1.28	0.324	15.6	3.6	1.90–6.78	<0.001***
Age	−0.091	0.095	0.92	0.91	0.76–1.10	0.336 ^NS^
BMI	−0.025	0.114	0.05	0.98	0.78–1.22	0.828 ^NS^
Gravida	−0.218	0.567	0.15	0.81	0.27–2.45	0.701 ^NS^

The participants were further categorised into three groups: (1) no hypotension, (2) hypotension not requiring vasopressors, and (3) hypotension requiring vasopressors. Patients requiring vasopressors demonstrated greater early MAP reductions compared with those with transient hypotension, supporting the association between severity of decline and intervention requirement.

Spinal anaesthesia for caesarean section resulted in a significant and transient decline in maternal MAP, most pronounced within the first 10 minutes after administration. While baseline maternal characteristics did not reliably predict hypotension, greater reductions in MAP were associated with vasopressor requirement, highlighting the relationship between the severity of haemodynamic decline and the need for intervention.

## Discussion

The present study demonstrates that spinal anaesthesia for caesarean section is associated with a substantial and predictable reduction in maternal arterial pressure, with 78 (78%) parturients developing hypotension and over one-third requiring vasopressor support. This high incidence is consistent with contemporary reports indicating that hypotension remains a frequent complication of spinal anaesthesia despite advances in monitoring and prophylaxis. A prospective observational study by *BMC Pregnancy and Childbirth* reported an incidence of 78.1%, nearly identical to that observed in our cohort, underscoring the persistent burden of spinal-induced hypotension across diverse healthcare settings [17]. Similarly, continuous non-invasive monitoring studies have shown that even with active management, maternal hypotension may occur in 40-55% of cases, depending on the MAP threshold used [18].

The high incidence of hypotension observed in this study may also be influenced by the absence of prophylactic vasopressor use, which is now recommended in many contemporary protocols. This should be considered when comparing incidence rates with studies employing preventive strategies.

Our findings also confirmed a rapid, time-dependent haemodynamic decline, with MAP reaching its nadir within five to 10 minutes of spinal administration, followed by a gradual recovery. This temporal pattern reflects the physiological consequences of acute sympathectomy and peripheral vasodilation and parallels previously described trajectories in obstetric anaesthesia literature. The early occurrence of the lowest MAP highlights the critical importance of vigilant haemodynamic surveillance during the initial post-block period and supports recommendations for proactive rather than reactive management.

Although demographic factors, such as age, BMI, and gravida, were evaluated, none independently predicted hypotension in our multivariable model. This contrasts with some observational data identifying high BMI and inadequate preload as risk factors. For example, the Mogadishu cohort reported higher BMI and low preoperative haemoglobin as significant predictors of hypotension [17]. However, our results align with emerging evidence suggesting that baseline characteristics alone may have limited discriminatory value for predicting individual haemodynamic responses. Recent research has therefore shifted toward physiological and functional markers. A prospective ultrasound-based study demonstrated that combining heart rate, left ventricular end-diastolic area, and velocity time integral changes more accurately predicted spinal-induced hypotension than any single parameter, highlighting the potential superiority of dynamic cardiovascular indices over static demographic variables [19]. Similarly, systematic review protocols evaluating pulse oximetry-derived indices reflect ongoing efforts to develop non-invasive predictive tools for early risk stratification [20].

A key observation in our study was that greater reductions in MAP (ΔMAP) were significantly associated with vasopressor requirement. However, this relationship reflects the severity of post-spinal haemodynamic change rather than an independent predictive factor, as ΔMAP is derived from the same physiological process that prompts intervention. Therefore, this finding should be interpreted as a marker of haemodynamic deterioration rather than a true preoperative or early predictive variable. This finding emphasises that the severity of haemodynamic depression itself, rather than preoperative characteristics, is associated with the need for pharmacologic support. These findings highlight the importance of early detection of haemodynamic decline and appropriate clinical response; however, this study did not evaluate specific monitoring strategies or treatment protocols. Contemporary trials comparing vasopressor strategies further support early, proactive therapy. Randomised comparisons have suggested that norepinephrine may preserve maternal and fetal circulation more effectively than phenylephrine while maintaining blood pressure stability [21]. Dose-response investigations in high-risk groups, such as preeclamptic patients, demonstrate that prophylactic norepinephrine infusions reduce hypotension incidence and decrease rescue requirements without increasing adverse outcomes [22]. Conversely, preeclamptic patients have been reported to experience comparatively less hypotension and attenuated haemodynamic fluctuations under spinal anaesthesia, likely due to baseline vasoconstriction and altered vascular responsiveness [23].

Beyond pharmacologic management, several investigators have explored technique-related strategies to mitigate hypotension. Height-adjusted dosing algorithms for intrathecal bupivacaine have been shown to reduce the spread of sensory block and lower the incidence of hypotension without routine prophylactic fluids or vasopressors [24]. Such approaches suggest that individualised anaesthetic dosing may complement haemodynamic monitoring and vasopressor use in minimising instability.

Although maternal hypotension has implications for neonatal outcomes, this study did not assess neonatal parameters, such as Apgar scores or cord blood gases. Therefore, conclusions regarding neonatal impact cannot be drawn from the present data and are included here only as contextual background.

Collectively, these results corroborate current evidence that spinal anaesthesia produces an early, transient, but significant decline in maternal arterial pressure. Although baseline maternal characteristics alone may not reliably identify at-risk patients, real-time haemodynamic changes, particularly the extent of MAP reduction, provide descriptive insight into intervention needs. The integration of continuous monitoring, early vasopressor administration, and individualised anaesthetic techniques may offer the most effective strategy to enhance maternal safety.

Several important intraoperative variables, including fluid administration, uterotonic use, blood loss, and sensory block height, were not included in the analysis. These factors may have influenced maternal haemodynamic responses and vasopressor requirement and should be considered when interpreting the findings.

Overall, this study provides local prospective data on serial MAP trends, quantifies the magnitude and timing of haemodynamic depression, and demonstrates that greater reductions in MAP are associated with vasopressor requirement, reflecting the severity of haemodynamic compromise. These findings contribute to ongoing efforts to provide descriptive insights into perioperative haemodynamic patterns in routine clinical practice.

Limitations

This study has several limitations. First, the single-centre design and modest sample size limit the generalizability of the findings. A formal sample size calculation was not performed, and the sample was based on feasibility, which may affect statistical precision. Second, haemodynamic data were recorded at predefined intervals rather than continuous beat-to-beat monitoring, which may have resulted in underestimation of transient hypotensive episodes. Third, the study did not use a standardised vasopressor protocol, nor was any prophylactic vasopressor infusion administered. This may have influenced both the incidence of hypotension and the threshold for intervention. Fourth, ΔMAP, the primary variable associated with vasopressor use, is a derived measure based on post-spinal haemodynamic changes. Therefore, its association with intervention reflects severity rather than true predictive capability, limiting its clinical applicability as an independent predictor. Fifth, important intraoperative confounders, such as fluid administration, uterotonic use, blood loss, and sensory block height, were not systematically recorded or analysed. Sixth, neonatal outcomes were not assessed, limiting the ability to evaluate the clinical impact of maternal hypotension on fetal well-being. Finally, advanced haemodynamic monitoring techniques and dynamic predictors were not included, which may have provided more robust insights into the mechanisms of spinal-induced hypotension.

## Conclusions

Spinal anaesthesia is consistently associated with an early decline in maternal MAP, and hypotension remains a common intraoperative occurrence during caesarean delivery. In this study, baseline maternal characteristics did not reliably identify patients at risk of hypotension. Greater reductions in MAP were associated with increased vasopressor requirements, reflecting the severity of haemodynamic compromise rather than being an independent predictor. These findings provide descriptive insights into perioperative haemodynamic patterns in routine clinical practice. However, interpretation should be cautious given the observational design and the absence of standardised intervention protocols.
